# MicroRNA-21 inhibitor sensitizes human glioblastoma cells U251 (PTEN-mutant) and LN229 (PTEN-wild type) to taxol

**DOI:** 10.1186/1471-2407-10-27

**Published:** 2010-01-31

**Authors:** Yu Ren, Xuan Zhou, Mei Mei, Xu-Bo Yuan, Lei Han, Guang-Xiu Wang, Zhi-Fan Jia, Peng Xu, Pei-Yu Pu, Chun-Sheng Kang

**Affiliations:** 1 Laboratory of Neuro-oncology, Tianjin Neurological Institute, Tianjin, PR China; 2 Tianjin research center of basic medical science, Tianjin medical university, Tianjin, PR China; 3 The First Department of Head and Neck Cancer, Tianjin Medical University Cancer Institute & Hospital, Tianjin, PR China; 4 School of Materials Science & Engineering, Tianjin University, Tianjin, PR China; 5 Department of Neurosurgery, Tianjin Medical University General Hospital, Tianjin, PR China

## Abstract

**Background:**

Substantial data indicate that the oncogene microRNA 21 (miR-21) is significantly elevated in glioblastoma multiforme (GBM) and regulates multiple genes associated with cancer cell proliferation, apoptosis, and invasiveness. Thus, miR-21 can theoretically become a target to enhance the chemotherapeutic effect in cancer therapy. So far, the effect of downregulating miR-21 to enhance the chemotherapeutic effect to taxol has not been studied in human GBM.

**Methods:**

Human glioblastoma U251 (PTEN-mutant) and LN229 (PTEN wild-type) cells were treated with taxol and the miR-21 inhibitor (in a poly (amidoamine) (PAMAM) dendrimer), alone or in combination. The 50% inhibitory concentration and cell viability were determined by the MTT assay. The mechanism between the miR-21 inhibitor and the anticancer drug taxol was analyzed using the Zheng-Jun Jin method. Annexin V/PI staining was performed, and apoptosis and the cell cycle were evaluated by flow cytometry analysis. Expression of miR-21 was investigated by RT-PCR, and western blotting was performed to evaluate malignancy related protein alteration.

**Results:**

IC(50) values were dramatically decreased in cells treated with miR-21 inhibitor combine with taxol, to a greater extent than those treated with taxol alone. Furthermore, the miR-21 inhibitor significantly enhanced apoptosis in both U251 cells and LN229 cells, and cell invasiveness was obviously weakened. Interestingly, the above data suggested that in both the PTEN mutant and the wild-type GBM cells, miR-21 blockage increased the chemosensitivity to taxol. It is worth noting that the miR-21 inhibitor additively interacted with taxol on U251cells and synergistically on LN229 cells. Thus, the miR-21 inhibitor might interrupt the activity of EGFR pathways, independently of PTEN status. Meanwhile, the expression of STAT3 and p-STAT3 decreased to relatively low levels after miR-21 inhibitor and taxol treatment. The data strongly suggested that a regulatory loop between miR-21 and STAT3 might provide an insight into the mechanism of modulating EGFR/STAT3 signaling.

**Conclusions:**

Taken together, the miR-21 inhibitor could enhance the chemo-sensitivity of human glioblastoma cells to taxol. A combination of miR-21 inhibitor and taxol could be an effective therapeutic strategy for controlling the growth of GBM by inhibiting STAT3 expression and phosphorylation.

## Background

Glioblastoma multiforme (GBM) is the most malignant form of human astrocytoma [1] and the median survival of GBM has remained less than one year over the past decade. Phosphates and tensin homolog (PTEN) located on chromosome ten, which encodes a cytoplasmic enzyme with both protein and lipid phosphates activity, is mutated, decreased or not expressed at chromosome 10q23 in 20~40% of malignant glioblastomas [2]. Loss of function PTEN leads to Akt activation by PI3K phosphorylation and results in poor prognosis of GBM [3,4]. Downstream activated Akt is associated with the RTKs (receptor tyrosine kinases), which include EGFR, IGFR, and VEGFR. These activated components of RTK pathways could further promote cell survival and anti-apoptotic reactions through phosphorylation and inactivation of downstream factors [5-7]. Thus, PTEN is a key checkpoint in the Akt signaling pathway and its dysfunction triggers RTKs-dependent oncogenesis.

Chemotherapeutic drugs are fundamental in cancer management and are responsible for most cases of adjuvant treatment in patients with GBMs after surgical procedures. Recently, much attention has been focused on the use taxol on glioma, both in experimental studies and in clinical trails [8]. However, the median overall survival did not increase in patients treated by concurrent chemoradiotherapy. Consequently, further studies that could enhance the therapeutic effect of taxol should be encouraged.

MicroRNAs (miRNAs), a newly discovered family of genes encoding small RNA molecules which bind through partial sequence homology to the 3'-untranslated regions (3'-UTRs) of target genes, play key roles in the regulation of gene expression [9]. Due to this unique feature, a single miRNA has multiple targets. Previous studies have shown that miRNA expression profiles provide valuable molecular signatures for different tissues and human cancers. Recent reports have identified miR-21 as the only miRNA that is overexpressed in nine types of solid tumors [10]. Accumulating evidence indicates that downregulation of miR-21 in glioblastoma cells leads to deregulation of these pathways, causing repression of growth, increased apoptosis, and cell cycle arrest, all of which could theoretically enhance the chemotherapeutic effects of cancer therapy [11].

In this study, we aimed to explore whether downregulating miR-21 could enhance the chemotherapeutic effect of taxol on human glioblastoma U251 (PTEN-mutant) and LN229 (PTEN wild-type) cells, using a poly (amidoamine) (PAMAM) dendrimer delivery system. PAMAM was employed as a carrier to deliver an miR-21 inhibitor to human glioblastoma cells to investigate the chemo-sensitivity of human glioblastoma U251 (PTEN-mutant) and LN229 (PTEN wild-type) cells to taxol. The 50% inhibitory concentration (IC(50)) values of taxol, determined by the MTT assay, were dramatically decreased in the cells transfected with the miR-21 inhibitor. It is worth noting that the miR-21 inhibitor additively interacted with taxol on U251cells and synergistically on LN229 cells. Furthermore, the miR-21 inhibitor significantly enhanced apoptosis in both U251 cells and LN229 cells and the invasiveness of the cells was obviously weakened compared with the single taxol chemotherapy or miR-21 inhibitor gene therapy. The cell cycle was arrested in G0/G1 and S phase.

Interestingly, the above data suggested that in both the PTEN mutant and wild-type GBM cells, miR-21 blockage increased the chemosensitivity to taxol. Thus, the miR-21 inhibitor might interrupt the activity of EGFR pathways, independent of PTEN status. Taken together, a combination of the miR-21 inhibitor and taxol could be an effective therapeutic strategy for suppressing the growth of GBM, independently of PTEN status.

## Methods

### Materials and Reagent

Human glioblastoma cell lines U251 and LN229 were obtained from the China Academia Sinica cell repository, (Shanghai, China). We obtained antibodies from Santa Cruz Biotechnology (Santa Cruz, CA, USA). The methanolic solution of PAMAM dendrimer (generation five) containing 128 surface amino groups (G5-PAMAM, G5D), and fluorescein isothiocyanate (FITC) were purchased from Sigma-Aldrich (St. Louis, MO). Semisynthetic taxol (T7191) was provided by Sigma-Aldrich (St. Louis, MO). The 2'-*O*-methyl (2'-OMe-) miR-21 inhibitors were chemically synthesized by Shanghai GenePharma (Shanghai, China). 2'-*O*-Me oligos were composed entirely of 2'-*O*-methyl bases and had the following sequences: miR-21 inhibitor: 5'-GTC CAC TCT TGT CCT CAA TG-3'; scrambled sequences were 5'-AAG GCA AGC UGA CCC UGA AGU-3'. The oligonucleotides were purified by a high-pressure liquid chromatography system, dissolved in diethylpyrocarbonate (DEPC) water, and frozen at -20°C.

### Cell Culture and transfection

The cells were maintained in Dulbecco's modified Eagle's medium (DMEM) (Gibco, USA) supplemented with 10% fetal bovine serum (Gibco, USA), 2 mM glutamine (Sigma, USA), 100 units of penicillin/ml (Sigma, USA), and 100 μg of streptomycin/ml (Sigma, USA), and incubated at 37°C with 5% CO_2_. Cells were seeded in 75-cm^2 ^flasks and incubated at 37°C in a fully humidified atmosphere with 5% CO_2_. Once the cells were 80% confluent, they were starved in DMEM with 1% FBS for 24 h and maintained in this low serum condition for the course of all treatments.

The G5 PAMAM dendrimers were first dialyzed against PBS (M.W. cut off 7000) for one day and then against deionized water for another day to remove the methanol. The miR-21 inhibitor solution (20 μmol/L) was incubated with G5-PAMAM solution as previously described [12]. For the combination treatment, cells were incubated with the inhibitor prior to the addition of taxol.

### RNA extraction and real-time PCR

The miRNA was isolated 72 hours after transfection with Ambion mirVana™ miRNA isolation kit (Ambion, USA). A nanodrop spectrophotometer (Gene, USA) was used to detect the concentration of total miRNA. Reverse transcription (RT) was conducted with the mirVana™ qRT-PCR miRNA detection kit (Ambion, USA) in a 10 μl reaction system, comprising 2 μl mirVana™ 5×RT buffer, 1 μl mirVana™ 1×RT primer, 25 ng total miRNA, 0.4 μl ArrayScript™ enzyme mix, and DDW (Deuterium Depleted Water) up to 10 μl. The RT reaction was performed at 37°C for 30 min and then 95°C for 10 min. Real-time PCR was carried out with the mirVana™ qRT-PCR miRNA detection kit (Ambion, USA) in 15 μl reaction: 2 μl mirVana™ 5×PCR buffer, 0.5 μl 50×ROX reference dye (Invitrogen, USA), 0.2 μl SuperTaq, 0.5 μl mirVana PCR primer (Ambion, USA), and DDW up to 15 μl. The amplification reaction was performed using MJ-real time PCR (BioRad, USA) and the protocol was performed for 40 cycles, comprising 95°C for 3 min, 95°C for 15 sec, 60°C for 30 sec. Both RT and PCR primers were purchased from Ambion. 5S was used for normalization. Relative quantification was conducted using amplification efficiencies derived from cDNA standard curves. Data were shown as fold change (2^-ΔΔCt^) and analyzed initially using Opticon Monitor Analysis Software V2.02 software (MJ Research, USA).

### Protein extraction and Western blotting

After the treatments, cells were lysed in a buffer composed of 50 mM Tris-HCl, pH 7.4, 0.1 mM phenylmethylsulfonyl fluoride (PMSF), and 5 mM EGTA for extraction of cellular proteins. The concentration of total proteins was determined colorimetrically using Coomassie-Plus protein assay reagent (Pierce, Rockford, IL, USA). The samples were mixed with an equal volume of 2× loading buffer [125 mM Tris-HCl, pH 6.8, 4% sodium dodecyl sulfate (SDS), 20% glycerol, 200 mM 1,4-dithio-dl-threitol (DTT), and 0.02% bromophenol blue], boiled for 5 min, and loaded (40 μg/lane) onto a 10% gradient gel for SDS-polyacrylamide gel electrophoresis (SDS-PAGE). After SDS-PAGE, the gels were blotted onto Immunobilon-P nylon membrane. The blots were blocked in 5% non-fat milk, 0.1% Tween, Tris-HCl, pH 7.8, for two hours at room temperature. The blots were then incubated with a specific primary IgG antibody for two hours at room temperature or overnight in a cold room, followed by alkaline horseradish peroxidase-conjugated secondary IgG antibody for one hour. Blots were developed using the enhanced chemiluminescence (ECL) reagents (Amersham Pharmacia, Buckinghamshire, UK) and visualized using the GeneGenius Imaging System (Syngene, Frederick, MD,USA).

### Cell viability assay

The cell viability was determined by the MTT (3- (4, 5-dimethylthiazole)-2, 5- diphenyltetrazoliumbromide) assay. Briefly, 10^4 ^cells/well were seeded in 96-well plates and allowed to attach overnight. The concentrations of free-taxol and miR-21 inhibitor were 6 mg/L and 20 μmol/L, respectively. The Scr-Oligo transfected cells were set as negative controls. Each group contained eight wells. On each day of five consecutive days, 20 μL of MTT (0.5 mg/mL) was added to each well and the cells were incubated at 37°C for 4 h. The reaction was then stopped by lysing the cells with 200 μL of dimethyl sulfoxide (DMSO) for 15 min. Quantification measurements (optical density) were obtained at a wavelength of 570 nm using spectrophotometric analysis. IC50 values were calculated from the linear regression line of the plot of percentage inhibition versus log inhibitor concentration.

### Cell Cycle Analysis

For cell cycle analysis by FCM (flow cytometry), transfected and control cells in the log phase of growth were harvested, washed with PBS, fixed with 90% ethanol overnight at 4°C, and then incubated with RNase at 37°C for 30 min. The nuclei of cells were stained with propidium iodide for an additional 30 min. A total of 10^4 ^nuclei were examined in a FACS Calibur flow-cytometer (Becton Dickinson, USA). Samples were analyzed by flow cytometry for the FL-2 area and DNA histograms were analyzed by Modifit software. Experiments were performed in triplicate. Results were presented as % of cell in a particular phase.

### Evaluation of cell apoptosis

To quantify drug-induced apoptosis, annexin V/PI staining was performed, and apoptosis was evaluated by flow cytometry analysis. Briefly, after treatment with the miR-21 inhibitor and the drug, both floating and attached cells were collected and subjected to annexin V/PI staining using an annexin V-FITC Apoptosis Detection Kit (BioVision, Palo Alto, CA), according to the manufacturer's protocol. The resulting fluorescence was measured by flow cytometry using a FACS flow cytometer (Becton Dickinson, San Jose, CA).

### Cell invasion assessment

Cell invasion abilities were examined using six-well transwell chambers and a reconstituted extracellular matrix membrane (Matrigel, USA). The cell invasion chambers were prepared by placing 100 μL of a 1:5 dilution of Matrigel onto the filter, and incubating the filter at 37°C for 30 minutes to allow Matrigel polymerization. Cells treated with free taxol, the miR-21 inhibitor or monk (scrambled sequences), or transfected by PAMAM or the miR-21 inhibitor combined with taxol, were removed from the culture flasks and resuspended at 5×10^5 ^cells/mL in serum-free medium. Two milliliters of each cell suspension was added to the upper chambers. The chambers were incubated for 48 h at 37°C in a humid atmosphere of 5% CO_2_/95% air. The filters were then fixed in 95% ethanol and stained with hematoxylin. The upper surfaces of the filters were scraped twice with cotton swabs to remove non-migrated cells. The experiments were repeated in triplicate wells, and the migrated cells were counted microscopically (400×) in five different fields per filter.

### Analysis of the combination effect between miR-21 inhibitor and anticancer drug

To analyze the combination effect between the miR-21 inhibitor and the anticancer drug taxol, the Zheng-Jun Jin method [13] was used. This method provides a "Q" value, according to which the combination effect between two drugs can be classified as an antagonistic effect (Q<0.85), an additive effect (0.85<Q<1.15), or a synergistic effect (Q>1.15). The formula is Q = Ea+b/(Ea+Eb-Ea×Eb), where Ea+b, Ea and Eb are the average effect of the combination treatment, the effect of the miR-21 inhibitor only, and the effect of taxol only, respectively.

### Statistical analysis

Results were analyzed using SPSS software 11.0 and compared using one-way analysis of variance (ANOVA) with Fisher's post hoc test. Data were presented as mean ± standard deviation (SD) of separate experiments (n ≥ 3). P values less than 0.05 were considered to be significant.

## Results

### miR-21 expression in U251 and LN229 cells treated with combination therapy

**Figure 1:**
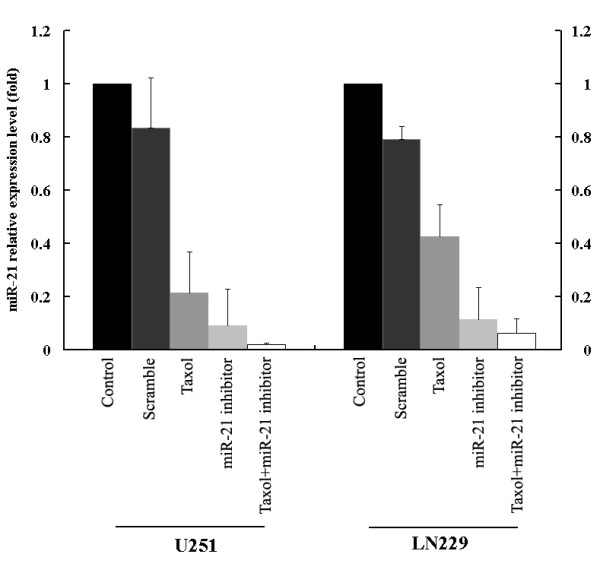
**Real time PCR analysis of miR-21 expression in glioblastoma cells treated with taxol and miR-21 inhibitor, alone or combination**. RT-PCR results showing significant knockdown of miR-21 expression in U251 and LN229 cells.

(2'-OMe-) antisense oligonucleotides were reported to knockdown miR-21 expression in human glioblastoma cells [14]. Regulation of miR-21 by the inhibitor was verified by RT-PCR, as shown in Fig.1. Transfection of the miR-21 inhibitor altered mir-21 levels relative to the control by 9.4-fold and 8.5-fold in U251 and LN229 glioblastoma cells, respectively. Interestingly, taxol alone also downregulated miR-21 expression. In both LN229 and U251 glioblastoma cells, the lowest level of miR-21 expression was achieved by treatment with the miR-21 inhibitor in combination with taxol therapy.

### miR-21 inhibitor increases the cytotoxicity of taxol on both U251 and LN229 cells

**Figure 2:**
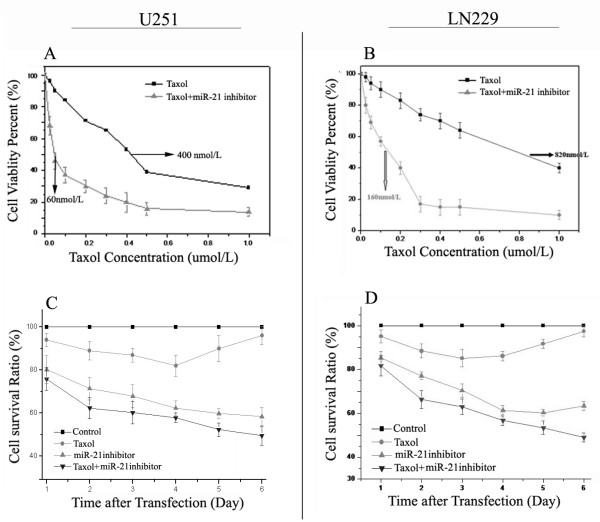
**Effect of the miR-21 inhibitor on the chemo-sensitivity of U251 and LN229 cells to taxol treatment**. The growth of U251 (Figure 2a) and LN229 (Figure 2b) cells were inhibited by the miR-21 inhibitor, taxol only, and the indicated combinations. The cells were treated with the miR-21 inhibitor complexed to PAMAM for 6 h at 37°C. The medium was then replaced with media containing various concentrations of taxol. After 72 h of incubation, an MTT assay was performed. Absorbance at 570 nm was normalized to the control (untreated cells) to determine cell viability. Each value represents the mean ±SD from triplicate determinations. An aqueous solution of taxol (circles) and miR-21 inhibitor-loaded PAMAM (triangle) was incubated with human glioblastoma U251 (Figure 2c) and LN229 (Figure 2d) cells for six days. Drug-induced decrease in cell numbers was measured using the MTT assay. Values represent the mean ± SD (*n *= 6 replicates).

For each experiment, dose-response curves were performed for each single chemotherapeutic drug and in combination with the miR-21 inhibitor. The result indicated that the miR-21 inhibitor can decrease the proliferation of both U251 and LN229 cells and increase the cells' sensitivity to taxol treatment. Fig 2A shows that the taxol concentration causing 50% growth inhibition (IC50) of U251 cells is 400 nmol/mL; whereas, in combination with the miR-21 inhibitor (20 μmol/L) the IC50 was 60 nmol/mL. Taxol can also increase the efficacy of the miR-21 inhibitor. For example, combination treatment reduced cell viability to 20% compared with 86% viability for miR-21 inhibitor gene therapy alone. In LN229 cells, combination treatment with 20 μmol/L of the miR-21 inhibitor reduced the IC50 of taxol from 820 to 160 nmol/L (Fig 2B). Analysis with SPSS software demonstrates statistically significant differences between any of the single drug treatments and the combination treatment, as indicated on Fig 2 (P < 0.01).

To evaluate the synergistic effect of miR-21 inhibitor-Loaded PAMAM with taxol on cell growth, we used the MTT assay to compare the growth of U251 and LN229 cells transfected with miR-21 inhibitor alone or with taxol. The measurements were made 72 h after transfection. The data (from triplicate samples) were analyzed for differences by unpaired, two-tailed *t *test. As indicated, taxol alone exhibited a moderate suppressive effect in the first three days of the MTT assay, resulting in maximal inhibition of 87% in U251 (Fig.2C), and 87% in LN229 glioblastoma cells (Fig. 2D). MiR-21 inhibitor-PAMAM complexes appeared to show better suppressive effects during the first three days of the MTT assay, however, combined treatment with the miR-21 inhibitor and taxol yielded a better effect on tumor growth suppression effect in the MTT assay, but the survival rate of both cells was still more than 60% on the sixth day of the MTT assay. Co-delivery of miR-21 inhibitor and taxol always maintained the best suppression effect during the entire MTT assay and resulted in maximal inhibition of 35% and 43% at 48 hours in U251 and LN229 glioblastoma cells, respectively.

### miR-21 inhibitor additively interacts with taxol on U251cells and synergistically on LN229 cells

To investigate the nature of the combination effect between the miR-21 inhibitor and the anticancer drugs on U251 and LN229 cells, the Zheng-Jun Jin method which was a useful approach to evaluate the combination effect among different drugs was performed to analyze the cytotoxicity data for antagonism, additivity, or synergy after cells are treated for three days. The Q value for LN229 cells was 1.32, indicating that synergistic effects appeared for the combination of the miR-21 inhibitor with taxol. On the other hand, the Q value for U251 cells was 0.97, indicating that the cytotoxicity data show additive effects after the cells were treated with the combination of the miR-21 inhibitor with taxol.

### Activities of the AKT pathway in human glioblastoma LN229 and U251 cell lines

**Figure 3:**
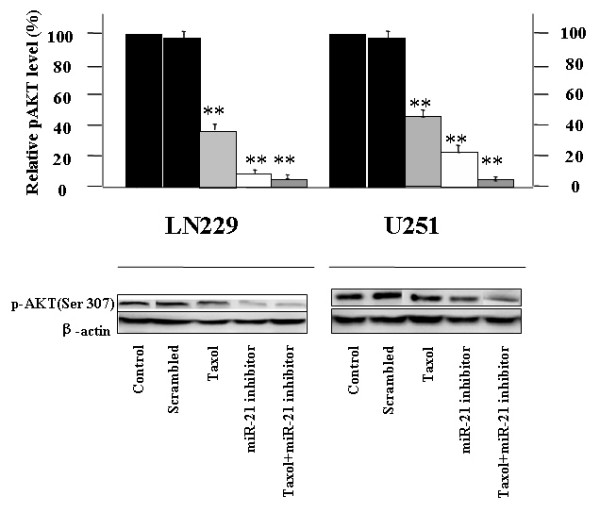
**Activity of the AKT pathway in human glioblastoma LN229 and U251 cell lines**. Representative Western blots showing the levels of p-AKT expression and the results of densitometric determinations. Significant differences from the control value are indicated by **p < 0.01.

Activation of the AKT pathway in malignant cells contributes to survival signaling [15]. We first examined the levels of phosphor-AKT expression in LN229 and U251 cells after treatment with the miR-21 inhibitor combination with taxol therapy (Fig. 3). The protein level determined by western blotting demonstrated that treatment of both LN229 and U251 cells with miR-21 inhibitor and taxol combination therapy could dramatically reduced the expression of phosphorylated Akt (p-Akt) significantly (*P *< 0.01) compared with either single taxol chemo-therapy or miR-21 inhibitor therapy alone.

**Figure 4:**
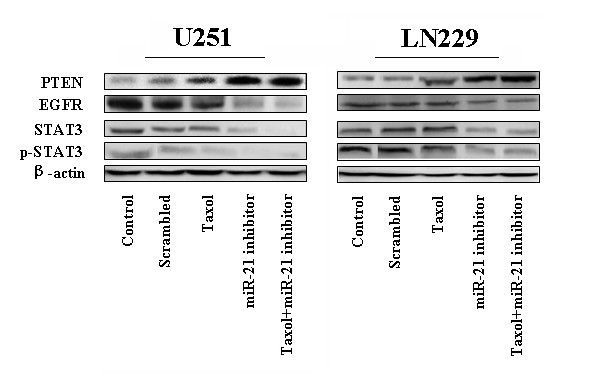
**Evaluation of the expression of PTEN, EGFR, STAT3, and p-STAT3 in human glioblastoma LN229 and U251 cell lines**. Western blot of protein extracts from cells treated with the miR-21 inhibitor or taxol, alone or combination. The expression of β-actin was examined to ensure uniform protein loading in all lanes.

And then we would like to further explore the effects of the miR-21 inhibitor and taxol on the key members of the AKT pathway. Overexpression of the gene encoding the epidermal growth factor receptor (EGFR) occurs commonly in glioblastoma, leading to activation of downstream kinases including phosphatidylinositol 3'-kinase (PI3K) and signal transducer and activator of transcription 3 (STAT3). Fig 4 shows Western blots for PTEN, a direct target of mir-21; EGFR, an initiator of the EGFR pathway; and STAT3 and phosphorylated STAT3, a nuclei co-factor of EGFR involved in cell-cycle progression and anti-apoptosis [16]. Actin was used as the control. Transfection of LN229 and U251cells with the miR-21 inhibitor or free taxol alone, caused varying degrees of increase in PTEN-related bands, reaching an approximately 5-fold increase for the miR-21 inhibitor and taxol combination treatment group. There is slight change in the protein level of EGFR in taxol treatment cells, on the contrast, 4.2-fold and 3.9-fold reduction was seen in combination therapy treated LN229 and U251 cells respectively... In addition, combination therapy also caused the notably down-regulation expression of both STAT3 and p-STAT3 compare to the both single treatment. U251 harbors the mutant form of PTEN, the direct target of miR-21; thus the data implies that miR-21 or taxol could be involved, in part, in the activities of EGFR pathways independently of PTEN status.

### miR-21 inhibitor and taxol induced apoptosis

**Figure 5:**
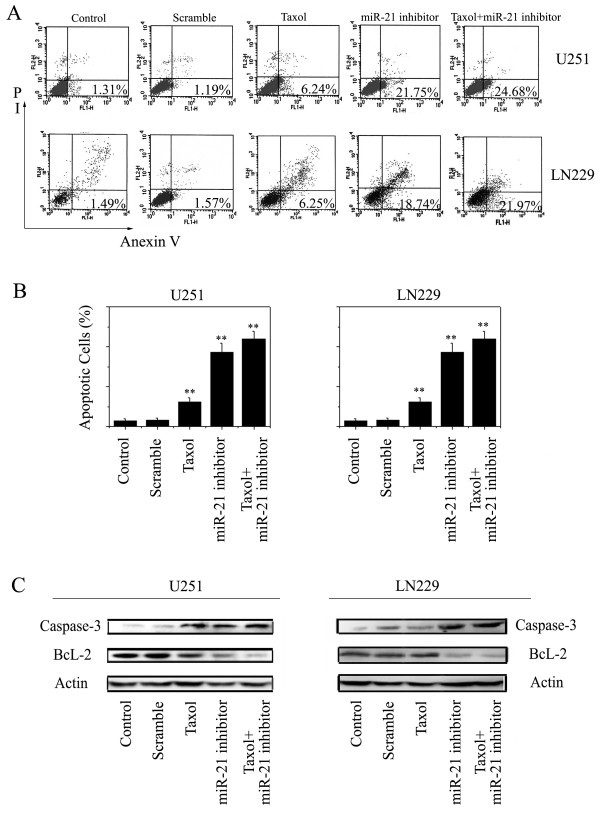
**The miR-21 inhibitor enhanced taxol induced apoptosis**. Flow cytometry analyses of propidium iodide-stained cells were performed in triplicate (Fig. 5A). Percentages of apoptotic cells are shown in the histogram (Fig. 5B). The effectiveness of the miR-21 inhibitor combined with taxol on BcL-2 and caspase-3 was analyzed by western blotting, with mock cells as controls (Fig. 5C). Antibodies against β-actin were used as a loading control to ensure uniform protein loading in all lanes.

FACS analysis was performed to detect DNA fragmentation in apoptotic cells following combined use of miR-21 inhibitor and taxol in U251 and LN229 human brain cancer cells (Fig. 5A). Untreated cells served as a negative control. Percentages of apoptotic cells are shown in the histogram (Fig. 5B). Compared with single taxol (6.24% and 6.25%, respectively) and miR-21 inhibitor (21.75% and 18.74%, respectively) treatment in U251 and LN229 cells, the combination of the miR-21 inhibitor and taxol therapy caused a significant (p < 0.05) increase amount (24.68% and 21.97%, respectively) of apoptotic death, suggesting that an additive induction of apoptosis developed in the cells co-infected with the miR-21 inhibitor and taxol.

Si et al [17] recently showed the knockdown of miR-21 inhibited tumor cell growth in vitro and in vivo by effecting an increase in apoptosis associated with downregulation of Bcl-2 expression, a potent anti-apoptotic regulatory factor. Preclinical studies have shown that ectopic expression of Bcl-2 confers resistance to several chemotherapeutic agents, including taxol [18]. In the current study, a significant decrease in the expression of Bcl-2 can be observed after treatment with taxol combined with the miR-21 inhibitor in U251 and LN229 cells (Fig.5C). The protein level of BcL-2 revealed an approximately 6-fold reduction in the miR-21 inhibitor alone treated cells, and an approximately 7.5-fold reduction in cells treated with the combination.

The in vitro sequence-specific functional inhibition of miR-21 in glioma cells leads to increased caspase levels, followed by cell death. Both miR-21 knockdown and taxol treatment alone depressed viability and caused caspase-3 upregulation in both cell lines, implicating apoptosis to be involved as a cell death mechanism [19]. However, marked additional caspase-3-associated cell death was observed for the combined treatment (P < 0.005). These findings indicate that, at least in vitro, knockdown of miR-21 before taxol administration sensitizes glioma cells for taxol cytotoxicity.

### Synergistic effects of miR-21 inhibitor and taxol on Cell cycle analysis

**Figure 6:**
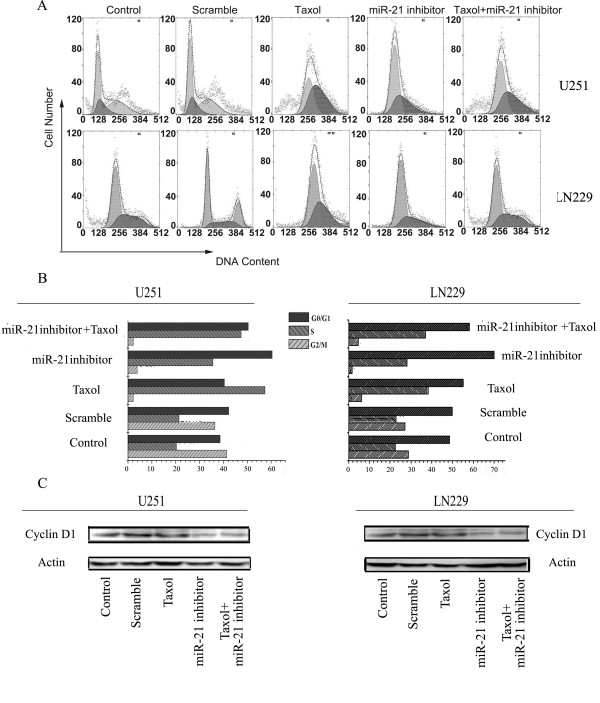
**miR-21 inhibitor and taxol induce G1 and S phase arrest on cell cycle distribution**. U251 and LN229 cells were treated with the miR-21 inhibitor and taxol alone or in combination, and cell cycle distributions were detected by Flow cytometry 48 h later (Fig. 6A). Percentages of cells in different phases of the cell cycle are shown in the histogram (Fig. 6B). Western blotting showing cyclin D1 activity in U251 and LN229 cells. (Fig. 6C) Representative Western blots showing altered levels of active cyclin D1 expression. The expression of β-actin was examined to ensure uniform protein loading in all lanes.

To better understand the synergistic effects on cell cycle progression, we exposed cells to the miR-21 inhibitor and taxol alone or in combination and evaluated changes in the cell cycle distribution by flow cytometry analysis (Fig.6A). Untreated cells served as negative controls. Treatment with taxol resulted in an increase in the population of cells that were in S phase. Fig.6B shows a representative experiment in which 20.3% of control U251 cells were in S phase, whereas taxol-treated cultures had 57.4% S-phase cells. Similarly, in Ln229 cells, taxol also caused an increase in S phase, from 22.5% to 38.2%.

Compared with control cells, the miR-21 inhibitor substantially and consistently increased the G1 population by 38.6% to 60.4% in U251 cells, by 48.7% to 70.1% in LN229 cells, indicating that miR-21 functions as a positive regulator of the G1-to-S transition. It is worth noting that the miR-21 inhibitor combined with taxol therapy produced both a greater percentage of G0/G1 and S phase cells, suggesting a synergistic effect of the combination on cell cycle progression.

The passage of cells through the cell division cycle is regulated by a family of kinases, the cyclin-dependent kinases (CDKs) and their activating partners, the cyclins. The G1/S phase transition is regulated primarily by D-type cyclins (D1, D2 or D3) in complex with CDK4/CDK6. No significantly alteration of cyclin D1 expression was observed with taxol alone (Fig.6C), suggesting that taxol alone does not produce any marked effect in the regulation of cell cycle in G0/G1 phase. The protein level of cyclin D1 revealed an approximately 4.4-fold reduction in U251 cells and a 4.2-fold decrease in LN229 cells, for treatment with the miR-21 inhibitor alone, and a 3.0-fold and 2.6-fold reduction, in the combined treatment U251 and LN229 cells, respectively.

### miR-21 inhibitor and taxol combination therapy regulate cell invasion

**Figure 7:**
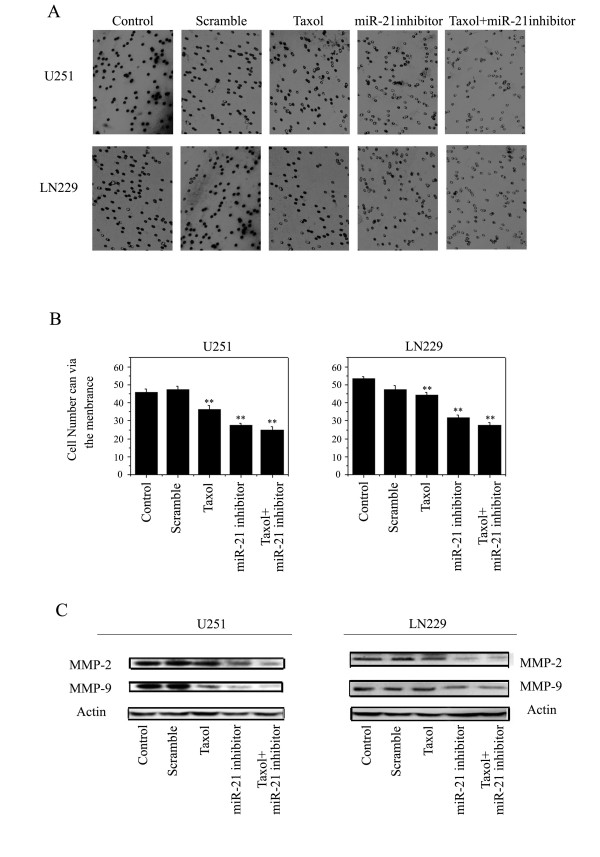
**Effects of miR-21 inhibitor and taxol on cell invasion ability in U251 and LN229 cell lines**. Cell invasion ability was assessed by a transwell assay after 48 h treatment with the miR-21 inhibitor and taxol, alone or in combination (Fig. 7A). The numbers of cells that could invade via the membrane are shown as a histogram (Fig. 7B). Western blotting validation of proteins (MMP-2 and MMP-9) downregulated by miR-21 inhibitor and taxol combination therapy in both U251 and LN229 cell lines (Fig. 7C). The expression of β-actin was examined to ensure uniform protein loading in all lanes.

To measure the effects the miR-21 inhibitor combined with taxol on glioma cell invasiveness, a better indication of glioma migratory and invasive properties in vitro, we employed a Transwell invasion assay. The system consists of two fluid-filled, stacked compartments, separated by a porous membrane filter coated with Matrigel. Cells were grown in the upper chamber and assessed for invasion through the Matrigel toward a chemo-attractant (10% serum) in the lower chamber. The number of invasive cells in cultures treated with the combination was significantly reduced relative to the control cells with out any treatment (Fig. 7A and 7B); a decrease of 46 to 25 in U251 cells and 54 to 28 in LN229 cells.

Accumulated data suggest that matrix metalloprotein (MMP) levels and activities are significantly elevated in human gliomas, which contributes to glioma cell invasion of the surrounding normal tissues, metastasis, and angiogenesis through cell surface ECM degradation [20]. In this respect, the levels of expression of MMP-2 and MMP-9 proteins in U251 and LN229 cells after combination treatments were also evaluated by western blotting (Fig.7C). Significantly decreased expression of MMP-2 and MMP-9 was observed after treatment with taxol combined with the miR-21 inhibitor in U251 and LN229 cells.

## Discussion

### miR-21 inhibitor sensitizes human glioblastoma cells to taxol

At present, cancer drug resistance is considered as a multifactorial phenomenon involving several major mechanisms, such as increased repair of DNA damage, reduced apoptosis, altered metabolism of drugs, and increased energy-dependent efflux of chemotherapeutic drugs that diminish the ability of cytotoxic agents to kill cancer cells [21]. MiRNA expression affecting multiple genes simultaneously, provided support for this hypothesis. This was evidenced by (a) downregulation of miR-451 leads to the increased metabolism of DOX [22]; (b) downregulation of miR-328 results in increased mitoxantrone sensitivity [23]; and (c) Overexpression of miR-221 and miR-222 in MCF-7 cells confers resistance to tamoxifen [24]. These finding supported the hypothesis that correction of altered expression of miRNA might have significant implications for therapeutic strategies aiming to overcome cancer cell resistance.

In this study, we demonstrated for the first time that the knockdown of miR-21 expression by a miR-21 inhibitor contributed to sensitizing human glioma cells to the anticancer drug taxol. MiR-21 was first implicated as an anti-apoptotic factor by the observation that knockdown of miR-21 increased apoptotic cell death in human glioblastoma cells [14]. It has been reported that miR-21 contributes to the malignant phenotype of tumor cells by blocking expression of critical apoptosis-related genes in human cancer cell lines. Despite the well-established role of miR-21 in GBM, the molecular mechanism of knocking down miR-21 in GBM chemotherapy remains largely unexplored.

Our dose-response data indicated that decreasing the miR-21 levels resulted in 6- and 5-fold increases in drug sensitivity (IC50), respectively, between inhibitor- and taxol-treated GBM cells. This demonstrated that the miR-21 inhibitor resulted in an increased sensitivity of glioma cells to taxol.

### miR-21 inhibitor enhances anti-proliferation effect of taxol to glioblastoma cells independent of PTEN status

Previous study proved that miR-21 could direct regulate PTEN tumor suppressor gene mRNA translation at post-transcriptional level in hepatocellular carcinoma and GBM cells [25]. Different genetic alterations of PTEN, including mutation, deletion, and translation suppression, could lead to aberrant EGFR pathway activation in GBM. Maier et al also analyzed the role of PTEN in invasion using the two highly infiltrative glioma cell lines U87MG (lacking PTEN function) and LN229 (PTEN wild-type) [26]. We deduced that knocking down miR-21 sensitized GBM to taxol through PTEN mRNA translation blockage.

Yet, it is worth noting that cytotoxicity data algorithm results indicated that the miR-21 inhibitor additively interacted with taxol on U251cells and synergistically on LN229 cells for MTT assay and additively for Annexin V/PI apoptosis assay in both GBM cell lines. Interestingly, the data of miR-21 inhibitor suppressed U251 GBM growth indicated there was an independent PTEN pathway although the exact mechanism was not clear. The above data suggested that both in the PTEN mutant and in the wild-type GBM cells, miR-21 blockage could increase the chemo-sensitivity to taxol. Chan et al reported that knocking down miR-21 could increase caspase3/7 activity similarly though in LN229 and U87 GBM cell that had different PTEN background [14]. Our previous research indicated that antisense miR-21 ODN could induce U251 and LN229 GBM cell apoptosis via attenuating EGFR signaling pathway. Besides, multiple cancer cell apoptosis or metastasis related genes including PDCD4[10], P53 signaling network[11], RECK[20], S-TRAIL[27] etc were validated to be miR-21's function targets in both brain tumors and other epithelium original human cancers. Presumably, miR-21 inhibitor mediated human GBM cell apoptosis effect in a one hit multiple target mechanism rather than directly inhibition of PTEN mRNA translation. Mild apoptosis induction difference of miR-21 inhibition in U251 and LN229 GBM cell suggested, compared to miR-21 blockage, PTEN wide-type or induction was a fine tune in the oncogenesis of GBM. And miR-21 suppression had clinical potential to enhance chemo-drug effect of chemotherapy in GBM patient with different PTEN genetic background.

EGFR has been a central focus of study in glioma due to its proposed role in the transformation and growth of glial tumors, and the fact that EGFR is the most commonly amplified gene in GBM. Activation of EGFR signaling plays a central role in GBM. AKT is the direct effector of EGFR downstream signaling; the expression of phosphorylated-AKT is the key factor representing the activities of EGFR pathways [28]. Both in U251 and in LN229 GBM cells, the miR-21 inhibitor could suppress the EGFR signaling pathway activity. From the data included in the manuscript, it's difficult to elucidate the exact mechanism that miR-21 inhibitor caused EGFR suppression in both PTEN mutant and wild type GBM. Bioinformatics analysis indicated that, EGFR mRNA didn't carry a miR-21 binding site (un-published data). Thus we deduced transcription inhibition might contribute to EGFR signaling pathway.

### Knocking down miR-21 enhances chemotherapeutic effect of taxol to glioblastoma cells via STAT3 inhibition and dephosphorylation

PI3K-AKT, Ras, and mitogen-activated protein kinases (MAPK), and receptor tyrosine kinases, including EGFR, contributed strongly to the growth and promotion of GBM. These diverse signaling pathways converge at specific transcription factors, including STAT3. STAT3 is constitutively activated in 60% of primary high-grade/malignant gliomas and the extent of activation correlates positively with glioma grade. The constitutive activation of STAT3 coexists with EGFR expression in 27.2% of primary high-grade/malignant gliomas [29]. Activated by EGFR or other RTKs, STAT3 proteins cooperate with other transcription factors to regulate expression of numerous malignancy related genes, including bcl-2, bcl-xL, mcl-1, p21^WAF1/CIP1^, MMP-9, and Cyclin D1. Stat3 was suppressed in the present study, consistent with it being predicted to be a miR-21 target by mathematic algorithm [30].

Fig. 3 shows that treatment of LN229 and U251 GBM cells with the miR-21 inhibitor or with taxol decrease the expression levels of EGFR, STAT3, and p-STAT3. In addition, the expressions of BCL-2, Caspase-3, Ki67, MMP2/9, and TIMP-1 were changed by miR-21 inhibition in U251 cell. These data can be explained by STAT3 inhibition and dephosphorylation.

Current therapy for malignant gliomas needs to seek novel targets and more effective, less toxic therapeutic strategies. The results from this study provide new rationales for novel combinational therapies using an miR-21 inhibitor to synergistically cooperate with taxol in PTEN-wt and PTEN-mutant patients. These findings also prompt the need for future evaluation of the therapeutic efficacy of EGFR/STAT3-based combinational therapy in targeting high-grade/malignant gliomas that overexpress miR-21.

## Conclusions

The above data suggested that in both the PTEN mutant U251 cell line and the PTEN wild-type LN229 cells, miR-21 blockage could increase the chemosensitivity to taxol. It is worth noting that the miR-21 inhibitor additively interacted with taxol on U251cells and synergistically on LN229 cells. Thus, the miR-21 inhibitor might interrupt the activity of EGFR pathways, independently of PTEN status. The miR-21 inhibitor enhanced the chemo-sensitivity of human glioblastoma cells to taxol and a combination of the miR-21 inhibitor and taxol could be an effective therapeutic strategy for suppressing the growth of GBM, independent of PTEN status.

## Competing interests

The authors declare that they have no competing interests.

## Authors' contributions

GXW and ZFJ carried out the cell culture and miR-21 transfection. LH performed miRNA extraction and Real Time PCR. PX was responsible for protein extraction and western blotting. MM participated in the collection of materials and YR and XZ were in charge of data collection, statistical analysis, and manuscript preparation. XBY, PPY, and CSK were responsible for the study design and for the preparation of the manuscript. All authors read and approved the final manuscript.

## Pre-publication history

The pre-publication history for this paper can be accessed here:

http://www.biomedcentral.com/1471-2407/10/27/prepub
